# Fertility intentions and perceived health status: A study of Polish migrants and non-migrants

**DOI:** 10.1016/j.jmh.2024.100264

**Published:** 2024-08-04

**Authors:** Nasim Ahamed Mondal

**Affiliations:** a Doctoral School of Social Sciences, University of Warsaw, Poland; b Centre of Migration Research (CMR), University of Warsaw, Poland

**Keywords:** Fertility intentions, Perceived health, Migrants, Non-migrants, Poland, The Netherlands

## Abstract

**Aims:**

This study aims, probably for the first time, to compare the fertility intentions of migrants and non-migrants of the same nationality at origin based on their self-rated health, wellbeing, and gender.

**Methods:**

The study utilized data from the *Families of Poles in the Netherlands (FPN) survey* and the *Generations and Gender Survey (GGS)*. This study used Blinder-Oaxaca decomposition models and generalized ordered logit models (gologit).

**Results:**

The findings reveal that both migrants and non-migrants who have good self-rated health and wellbeing are more likely to express a desire to have children in the short-term. The impact of perceived health on the intention to have children is significant for both female and male irrespective of their migration status. Additionally, the average fertility intentions score is higher for Polish migrants residing in the Netherlands compared to Polish non-migrants residing in Poland across the models. The study emphasizes the role good perceived health plays in shaping fertility intentions.

## Highlights

• Good perceived health boosts childbearing intentions in both migrants and non-migrants.
• Average fertility intentions score is higher for migrants compared to non-migrants.
• Perceived health's impact on childbearing intentions is stronger for both sex.

## 1 Introduction

The fertility of both migrants and non-migrants has long been a significant subject of study in the field of demography (Carlsson, 2022; Impicciatore et al., 2020; Mussino and Cantalini, 2022; Stonawski et al., 2016; Tønnessen, 2020). In the European context, this topic has gained renewed attention primarily due to the continuing prevalence of low fertility rates in most European nations since the 1990s (as noted by Kohler et al., 2002), which have persisted after the Great Recession (as discussed by Dantis and Rizzi, 2020 and; Matysiak et al., 2021). Fertility determinants such as income, educational attainment and employment have been closely examined as they influence the amount of time and money available to individuals (Berrington et al., 2015; d'Albis et al., 2017; Gatta et al., 2022; Jalovaara et al., 2019; Lappegård and Rønsen, 2005). Similarly, one's perceived health can be considered a factor that either aids or impedes fertility intentions. If someone feels their health is not good, it can make them less prepared to become a parent. They might worry about not being able to provide for a family or enjoy doing things together as a family. Feeling physically unfit can also make them doubt their ability to handle the demands of raising a child, like having enough energy to care for them and help them grow up well. However, despite its importance, perceived health has been insufficiently studied as a determinant of fertility.

The connection between an individual's perceived health and fertility has received little attention in demographic research, particularly in countries where health standards are high (Alderotti and Trappolini, 2022; Mynarska and Wróblewska, 2017). Evidence of health issues hindering fertility in such countries is mostly limited to medical studies. Moreover, studies exploring the relationship between perceived health and fertility intentions are few in number. Some studies explored fertility desires and intentions in different ways, albeit they did not cover how perceived health influences fertility intentions. One study explored fertility desires and intentions among US women by disability status (Bloom et al., 2017), and another investigated the timing of fertility intentions (Dommermuth et al., 2011). Fiori et al. (2017) made a comparative study of fertility intentions among men and women in Italy and Britain. A recent study compared the fertility intentions of migrant and native Italian women (Mussino et al., 2023). None of these studies focused on how perceived health influences fertility intentions. Based on literature search in the field of migration and fertility, one study has focused on how perceived health impacts the fertility intentions of migrant (-only) subgroups (Alderotti and Trappolini, 2022). Additionally, one study employed four distinct health indicators to examine whether women (only) with disabilities or various health issues tend to restrict their parental plans. The findings indicate that each health factor played a significant role in women's intentions to have a child within the next three years (Mynarska and Wróblewska, 2017). Another study was identified that explored intentions to remain childless among native Poles (Mynarska and Brzozowska, 2022). However, to the best of my knowledge, no investigation has been conducted in any settings including the Polish context to explore how perceived health is associated with fertility intentions among both male and female migrants and non-migrants together.

This study therefore aimed to remedy the lack of research about the relationship between perceived health and fertility intentions of Polish migrants and non-migrants of the same nationality at origin. The main objective of this investigation was to examine how perceived health is linked to fertility intentions of both male and female migrant and non-migrant populations. The study utilised two datasets – from *Families of Poles in the Netherlands* for Polish migrants residing in the Netherlands and from *Generations and Gender Survey* for Polish non-migrants – to assess whether perceived health influenced their short-term fertility intentions.

To achieve this goal, the association between SRH and subjective wellbeing as perceived health indicators and fertility intentions was examined. This study focused on Poland for two main reasons. Firstly, since Poland's accession to the EU in 2004, a significant number of Poles have emigrated, primarily for work, to other European countries (Fihel and Okólski, 2019; Kaczmarczyk and Okólski, 2008). Researchers have explored various aspects of Polish migrant groups, such as the factors influencing their choice of the Netherlands (Pszczółkowska, 2024), their decisions to return to Poland from the Netherlands (Jancewicz and Markowski, 2021), childbearing and parental decisions of intra EU migrants (Kloc-Nowak, 2018) and the experiences of discrimination and unfair treatment faced by newly arrived Poles in the Netherlands and other EU member states (Gul-Rechlewicz, 2020) etc. However, to the best of my knowledge, study on the role of perceived health on fertility intentions of Polish migrants is yet to be carried out. Secondly, this study utilizes data from the *Families of Poles in the Netherlands survey*, which exclusively targets Polish migrants, the largest immigrant group in the Netherlands (Statistics Netherlands, 2022). This dataset allows for a unique comparison of the fertility intentions of migrants and non-migrants of the same nationality at origin, based on their Self-rated health (SRH), wellbeing, and gender. The decision to investigate fertility intentions rather than actual fertility outcomes was mainly due to data constraints, as studying the relationship between perceived health and fertility outcomes of migrants and non-migrants would require high-quality longitudinal data that is not available for Poland or for several other European countries. Nonetheless, fertility intentions offer a reliable measure of future fertility behaviour (Rindfuss et al., 1988; Schoen et al., 1999). Investigating them also helps understand if people have parenthood in plans and what their upper limit of the number of children could be.

## 2 Literature review

Research in the medical field indicates that individuals who have health issues are likely to have a lower desire to have children (Cvancarova et al., 2009; McGrath et al., 1999) and tend to have fewer offspring compared to healthy individuals (Chen et al., 2001; Langeveld et al., 2002). The mechanisms by which health problems may affect fertility have been widely studied. One of them is that treating a health issue may lead to infertility problems (Dow and Kuhn, 2004). In women, the physical aspect is especially relevant to the link between health and fertility because pregnancy is a physically demanding process. Women may be worried about the potential impact of pregnancy and motherhood on their illness or disease and about the welfare of their child (Dow and Kuhn, 2004; Fair et al., 2000; Langeveld et al., 2002). Women may also be concerned that their health problems could make pregnancy more difficult or cause complications (Drew, 2003) and that they (this factor also concerns men) could pass on their problem to their offspring (Katz, 2006). However, the physical aspect is also important for men's health and fertility. For instance, BMI and physical fitness can affect men's sexual function and sperm quality (Cheng and Ng, 2007; Hammoud et al., 2008). Obesity is linked to reduced fertility and sub-fecundity in both genders (Ramlau-Hansen et al., 2007). Moreover, poor physical health and its associated limitations may influence fertility by hindering the future parents' ability to care for their children (Katz, 2006).

There is limited evidence available on the relationship between mental health (also referred to as wellbeing) and fertility. Studies suggest that individuals with mental health issues tend to have fewer children (McGrath et al., 1999) and report infertility issues more frequently than those with good mental health (Bhongade et al., 2015). Earlier research (Bartel and Taubman, 1986; Nijs et al., 1985) examined the role of mental health and psychological stress in causing infertility. However, more recent studies, such as HMHL (2009), propose that negative mental health may be a consequence rather than a cause of infertility. Most of the existing studies focus mainly on the impact of infertility on wellbeing (Luk and Loke, 2015; Monga et al., 2004; Schmidt, 2010).

Since this study concerns migrants, point to be remembered that studies found discrimination is associated with perceived health (Wanner and Pecoraro, 2023; Wiking et al., 2004). Wanner and Pecoraro (2023) found that more than half of the foreign-origin population report having experienced prejudice or discrimination in their study. They also highlighted that these experiences of perceived discrimination occur in various aspects of professional and private life, particularly in public settings (such as shops and leisure activities), as well as in the workplace and educational environments.

The connection between perceived health and fertility has not received much attention in demographic research, particularly in middle and high-income countries where health standards are relatively good. However, health problems may impact fertility not only through the direct channels suggested by medical literature, but also indirectly. For instance, poor health can reduce one's ability to work, negatively affecting labour force participation and wages, which in turn could influence fertility (Alderotti et al., 2021). Health issues can also hinder the possibility of entering a stable relationship, the lack of which is one of the primary paths to childlessness in developed countries (Mynarska et al., 2015; Tocchioni, 2018). One of the few non-medical studies exploring the relationship between health and fertility, Holton et al. (2011), examined the childbearing expectations and outcomes of Australian women and found that health problems were a significant factor affecting childbearing, often preventing women from achieving their desired fertility outcomes. Frisco and Weden (2013) also observed a similar pattern in a sample of American women, where obesity in their twenties was associated with a higher likelihood of underachieving fertility intentions. Furthermore, Barclay and Kolk (2020) investigated the link between men's health and fertility in Sweden and discovered a strong relationship between anthropometric measures (such as BMI, physical fitness, and height) and total fertility, even after adjusting for education and income. To my knowledge, only one study (Alderotti and Trappolini, 2022) has explored the connection between perceived health and fertility intentions among migrant subpopulations, finding that poor self-rated health negatively impacts intentions to have children. I have not found any literature that compared the fertility intentions of migrants and non-migrants of the same nationality at origin based on their Self-rated health (SRH) and wellbeing.

## 3 Research hypotheses

The primary objective of this investigation was to investigate the relationship between perceived health and fertility intentions among Polish migrants and non-migrants of both genders using two different measures of health: SRH and wellbeing, collectively referred to as perceived health in this study. Separate analyses were conducted for each health measure, and the study was stratified by gender, in line with previous research on perceived health and fertility intentions (Alderotti and Trappolini, 2022). The following two hypotheses were tested:

My first hypothesis was that the relationship between perceived health and intentions to have children would be positively stronger among migrant Poles (H1). According to previous research, the health status of immigrants in wealthy nations is better than that of natives in the origin country (such as the study by Wallace et al., 2019), the probable reasons being selective migration (as proposed by Lee, 1966; McDonald and Kennedy, 2004), cultural factors (as suggested by Lee et al., 2013), and healthy behaviours among migrants (as discussed by Razum et al., 2000). We also know that poor health leads to negative fertility intentions (Alderotti and Trappolini, 2022).

My second hypothesis was that there would be a stronger positive association between perceived health and fertility intentions among women than among men for both migrants and non-migrants (H2). This is because women often pay closer attention to their health when making fertility decisions. Since pregnancy and childbirth involve significant physical demands, they tend to prioritize feeling healthy before planning to conceive. Women might also have worse SRH declarations because they are more aware and self-concerned (Boerma et al., 2016).

## 4 Materials and methods

### 4.1 Data set and variables

This investigation studied the relationship between perceived health and fertility intentions of Polish migrants and non-migrants of both genders. Data from *Families of Poles in the Netherlands* (FPN, Wave-1) was used for Polish migrants in the Netherlands (Karpinska, et al., 2016) and from *Generations and Gender Survey* (GGS, Wave-2) for Polish non-migrants (Gauthier et al., 2018). Both surveys were conducted in 2014-15. The FPN survey sample is representative of adult Polish-born persons living in the Netherlands. In the Netherlands, newcomers must register in the municipality where they want to live if their stay is to be longer than four months. The population registers were used to select a sample of adults (via simple random sampling, offering national coverage) who were born in Poland, had at least one Polish parent and first registered in the Netherlands between January 2004 and August 2014. The 2015 questionnaire of the *Generations and Gender Survey* was the blueprint for the FPN survey (Karpinska and Dykstra, 2019).

### 4.2 Outcome variable

The intention to have a/another child in the next 3 years was the study's primary outcome variable. The research was based on two different datasets. In FPN, the possible answers (5 categories) were “definitely not”, “probably not”, “unsure”, “probably yes” and “definitely yes”. In GGS, the possible answers (4 categories) were “definitely not”, “probably not”, “probably yes” and “definitely yes”. Individuals who answered “unsure” were excluded from the analysis for consistency between the two data sets.

### 4.3 Predictor variables

The primary independent variables were SRH and wellbeing, as two indicators of perceived health in this study. SRH was derived from a single question (i.e. "How is your health in general?") with five possible answers: "very good", "good", "fair", "bad" and "very bad". In the analyses, I used a dichotomous variable by coding "very good" and "good" as 0 and all other answers as 1. SRH is commonly utilized in various studies as a reliable indicator for forecasting morbidity, healthcare service utilization, mortality rates and general health status (Alderotti and Trappolini, 2022; Chandola and Jenkinson, 2000; Jylhä, 2009). However, certain authors (Assari et al., 2016; Woo and Zajacova, 2017) have recently emphasised the importance of exercising caution while using SRH to assess health disparities among different racial and ethnic groups, since people from diverse migrant backgrounds may assess their health in varying ways (Agyemang et al., 2006; Lubbers and Gijsberts, 2019).

For wellbeing, I created an index based on six questions present in both the datasets. All six questions were the same in both datasets, allowing better comparative analysis of Polish migrants and non-migrants. To ensure the same order (where a low score indicates better wellbeing), all responses were recoded to have the same direction. Possible scores for wellbeing varied between 6 and 18, with a lower score indicating better wellbeing. The wellbeing was set at 0 (good) if the respondent had a wellbeing index score below or equal to 12. The wellbeing does not have a uniform definition, and it is common to use it interchangeably with terms like quality of life, happiness, life satisfaction, and welfare, though some fields make subtle distinctions between them (Ali, 2010; Allin, 2007).

### 4.4 Control variables

To create profiles of individuals (migrants and non-migrants separately) and compute the discrete effects, the following six variables have been used for migrants, while the first four variables have been used for non-migrants: First, age at the interview was categorised into "21-30 years", "31-40 years" and "41-49 years". Second, the activity of the respondents was classified as, "Employed", "Self-employed", "Unemployed" and "Other". Third, education was classed as "ISCED 0-2", "ISCED 3-4" and "ISCED 5-6". It was followed by the parent status categorised into "No child" and "Having a child". Fifth, the reason for migration was divided into two categories: "Family migrants" and "Non-family migrants" followed by the duration of staying was categorised into "Up to 5 years" and ">5 years".

### 4.5 Modelling strategy

This study used generalized ordered logit models (Peterson and Harrell Jr., 1990) to examine the link between perceived health and fertility intentions. I chose the generalized ordered logit model (gologit) over the classic ordered logit model because the data showed that the proportional odds assumption failed to be met. I did not switch to the multinomial logistic model as it is less concise, more complex to interpret, and does not consider category ordering. Instead, I opted for the partial proportional odds gologit model, which relaxes the parallel-lines constraint only for variables where it is not reasonable (for details Williams, 2016). I used STATA16 and the gologit2 package (Williams, 2006) to run the analyses, and reported the discrete effects to examine the impact of SRH and wellbeing on short-term fertility intentions of Polish male and female migrants and non-migrants. Before the final model was run, multicollinearity between variables was tested. The distributions of dependent, independent and control variables for both data sets are given in the appendices (Table A1).

The gologit2 is a STATA user-written program that fits generalized ordered logit models for ordinal dependent variables. The gologit model can be written as:

$$P\left(Yi\;>\;j\right)=\;g\left(X\beta j\right)=\frac{\exp\left(\alpha j\;+\;Xi\beta j\right)}{1\;+\;\left\{\text{exp}\left(\alpha j+Xi\beta j\right)\right\}},\;j=\;1,\;2,\;.\;.\;.,M\;-\;1,$$

where M is the number of categories of the ordinal dependent variable. From the above, it can be determined that the probabilities that Y will take on each of the values 1, . . ., M are equal to

P(Yi=1)=1−g(Xiβ1)

P(Yi=j)=g(Xiβj−1)−g(Xiβj)j=2,...,M−−1

P(Yi=M)=g(XiβM−1)

I run four Blinder-Oaxaca decomposition models on ordinal dependent variable. Model 1 is the crude results of the impact of SRH on fertility intentions; Model 2 is the adjusted results of the impact of SRH on fertility intentions by controlling for age, sex, activity, education and parent status of the respondents. Similarly, Model 3 is the crude results of the impact of wellbeing on fertility intentions; Model 4 is the adjusted results of the impact of wellbeing on fertility intentions by controlling for age, sex, activity, education and parent status of the respondents.

A decomposition of a outcome variable for a linear case is not similar to the nonlinear (NL) case, because the conditional expectations, E(Yig|Xig), may differ from $\bar{Xg\;}\;\hat{\beta g}$. Therefore, we can write in terms of conditional expectations to obtain a general version of the Blinder–Oaxaca decomposition:

$$\Delta \frac{NL}{A}=\left\{E\beta A\;\left(YiA|XiA\right)-E\beta A\left(YiB|XiB\right)\right\}+\left\{E\beta A\;\left(YiB|XiB\right)-E\beta B\left(YiB|XiB\right)\right\}$$

where Eβg(Yig|Xig) refers to the conditional expectation of Yig, and Eβg(Yih|Xih) refers to the conditional expectation of Yih evaluated at the parameter vector βg with g,h=(A,B) and g≠h. For more details, one can have a look at Sinning's paper (2008).

Since my dependent variable is an ordinal, here is the sample counterpart

$\frac{1}{Ng}\sum_{i=1}^{N}[\left\{\wedge \left(\hat{{\;}_{\mu 1}}-Xig\hat{{\;}_{\beta g}}\right)-\Lambda \left(-Xig\hat{\beta g}\right)\right\}+2\;\left\{\Lambda \left(\hat{{\;}_{\mu 1}}-Xig\hat{{\;}_{\beta g}}\right)-\Lambda \left(\hat{{\;}_{\mu 1}}-Xig\hat{{\;}_{\beta g}}\right)\right\}+\ldots +J\{1\;-\;\Lambda (\hat{{\;}_{\mu J}-1}-Xig\;\hat{\_\beta g)\}],}$ where J is the number of possible outcomes and $\hat{\mu 1,}\ldots ,\hat{\_\mu J-1}$ are the estimated threshold values of ologit.

## 5 Results

This study focuses solely on the relationship between perceived health and fertility intentions. Thus, I only report the results as regards the effect of each of the two measures of perceived health on fertility intentions, separately for each subgroup analysed. Table 1 shows the contribution of selected predictors on intentions to have a/another children difference between Polish migrants living in the Netherlands and non-migrants living in Poland. Table 2 illustrates the discrete effects of how migration status affects the fertility intentions of both migrants and non-migrants. Table 3, Table 4 show the discrete effects of reported SRH and wellbeing on the probability of giving a specific answer to the question about fertility intentions by men and women.

**Table 1:** Oaxaca decomposition for ordinal dependent variable: contribution of selected predictors on intentions to have a/another children difference between Polish migrants living in the Netherlands and non-migrants living in Poland, 2014-15.

Results	Model 1 (n = 5426)				Model 2 (n = 5426)				Model 3 (n = 5426)				Model 4 (n = 5426)			
	Coef.	% contribution	[95 % Conf.Interval]		Coef.	% contribution	[95 % Conf.Interval]		Coef.	% contribution	[95 % Conf.Interval]		Coef.	% contribution	[95 % Conf.Interval]	
Omega (Migrants)= 1																
Char	0.0079	1.26	-0.0034	0.0192	0.2593***	48.71	0.2146	0.3040	-0.0284**	-4.90	-0.0530	-0.0039	0.2446***	46.25	0.1914	0.2979
Coef	0.6193***	98.74	0.5154	0.7233	0.2730***	51.29	0.2130	0.3331	0.6085***	104.90	0.5382	0.6790	0.2843***	53.75	0.2023	0.3664
Omega (Non-migrants)= 0																
Char	0.5003***	79.76	0.2998	0.7008	1.071***	201.32	0.8940	1.2496	0.5715***	98.52	0.4857	0.6575	1.0378***	196.20	0.8040	1.2716
Coef	0.1270	20.24	-0.1560	0.4100	-0.5394***	-101.32	-0.7416	-0.3372	0.0086	1.48	-0.0362	0.0533	-0.5088***	-96.2	-0.7718	-0.2459
Raw	0.6272***	100	0.5220	0.7326	0.5323***	100	0.4666	0.5982	0.5801***	100	0.5163	0.6440	0.5289***	100	0.4480	0.6100

Sources: Author's elaboration after appending Families of Poles in the Netherlands (FPN) data (Karpinska, et al., 2016) and Generations and Gender Survey (GGS) data (Gauthier et al., 2018). Model 1 is the crude results of the impact of SRH on fertility intentions; Model 2 is the adjusted results of the impact of SRH on fertility intentions by controlling for age, sex, activity, education and parent status of the respondents. Similarly, Model 3 is the crude results of the impact of Wellbeing on fertility intentions; Model 4 is the adjusted results of the impact of Wellbeing on fertility intentions by controlling for age, sex, activity, education and parent status of the respondents.

**Table 2:** Generalized ordered logistic model for fertility intentions: migrants and non-migrants. Discrete effects are reported.

Intention to have children	Margin	*P*>z	[95 % Conf. Interval]	
Definitely not (Migrants)	0.310	0.000	0.280	0.341
Definitely not (Non-migrants)	0.544	0.000	0.530	0.558
Probably not (Migrants)	0.364	0.000	0.350	0.379
Probably not (Non-migrants)	0.302	0.000	0.290	0.315
Probably yes (Migrants)	0.231	0.000	0.209	0.253
Probably yes (Non-migrants)	0.116	0.000	0.108	0.124
Definitely yes (Migrants)	0.094	0.000	0.081	0.107
Definitely yes (Non-migrants)	0.038	0.000	0.034	0.042

Sources: Author's elaboration after appending Families of Poles in the Netherlands (FPN) data (Karpinska, et al., 2016) and Generations and Gender Survey (GGS) data (Gauthier et al., 2018), (n = 5426).

**Table 3:** Generalized ordered logistic model for fertility intentions: female and male migrants. Discrete effects are reported. Profile: Middle-aged (31-40 years old) and moderately educated (ISCED 3‒4) family migrants who are childless, employed, and have been living in the Netherlands for more than 5 years.

Self -rated Health (SRH)	Migrants							
	Female (*n* = 536)				Male (*n* = 308)			
Intention to have children	Margin	P>z	[95 % Conf. Interval]		Margin	P>z	[95 % Conf. Interval]	
Definitely not (Good SRH)	0.284	0.000	0.178	0.390	0.110	0.055	-0.003	0.223
Definitely not (Bad SRH)	0.404	0.000	0.261	0.548	0.093	0.087	-0.013	0.200
Probably not (Good SRH)	0.257	0.000	0.206	0.308	0.102	0.016	0.019	0.185
Probably not (Bad SRH)	0.265	0.000	0.217	0.312	0.090	0.036	0.006	0.174
Probably yes (Good SRH)	0.293	0.000	0.224	0.362	0.340	0.000	0.234	0.445
Probably yes (Bad SRH)	0.227	0.000	0.146	0.309	0.323	0.000	0.189	0.457
Definitely yes (Good SRH)	0.165	0.000	0.091	0.240	0.448	0.002	0.169	0.726
Definitely yes (Bad SRH)	0.104	0.001	0.045	0.162	0.494	0.002	0.185	0.803
Wellbeing	Female (*n* = 536)				Male (*n* = 308)			
Definitely not (Good Wellbeing)	0.295	0.000	0.188	0.402	0.096	0.065	-0.006	0.198
Definitely not (Bad Wellbeing)	0.428	0.000	0.269	0.586	0.128	0.072	-0.012	0.268
Probably not (Good Wellbeing)	0.260	0.000	0.210	0.309	0.092	0.024	0.012	0.172
Probably not (Bad Wellbeing)	0.262	0.000	0.212	0.312	0.114	0.017	0.020	0.208
Probably yes (Good Wellbeing)	0.287	0.000	0.218	0.356	0.327	0.000	0.205	0.450
Probably yes (Bad Wellbeing)	0.215	0.000	0.128	0.302	0.352	0.000	0.260	0.445
Definitely yes (Good Wellbeing)	0.158	0.000	0.087	0.229	0.485	0.001	0.198	0.771
Definitely yes (Bad Wellbeing)	0.095	0.001	0.036	0.154	0.405	0.008	0.107	0.703

Source: Authors’ elaboration on Families of Poles in the Netherlands (FPN) data (Karpinska, et al., 2016).

**Table 4:** Generalized ordered logistic model for fertility intentions: female and male non-migrants. Discrete effects are reported. Profile: Middle-aged (31-40 years old) and moderately educated (ISCED 3‒4) non-migrants who are childless and employed.

Self-rated Health (SRH)	Non-migrants							
	Female (*n* = 2709)				Male (*n* = 1873)			
Intention to have children	Margin	P>z	[95 % Conf. Interval]		Margin	P>z	[95 % Conf. Interval]	
Definitely not (Good SRH)	0.374	0.000	0.318	0.431	0.204	0.000	0.171	0.237
Definitely not (Bad SRH)	0.431	0.000	0.361	0.502	0.225	0.000	0.179	0.271
Probably not (Good SRH)	0.362	0.000	0.338	0.386	0.398	0.000	0.374	0.423
Probably not (Bad SRH)	0.348	0.000	0.319	0.378	0.407	0.000	0.381	0.433
Probably yes (Good SRH)	0.190	0.000	0.157	0.224	0.293	0.000	0.260	0.327
Probably yes (Bad SRH)	0.162	0.000	0.126	0.197	0.275	0.000	0.233	0.317
Definitely yes (Good SRH)	0.073	0.000	0.055	0.092	0.104	0.000	0.083	0.125
Definitely yes (Bad SRH)	0.059	0.000	0.041	0.076	0.093	0.000	0.069	0.117
Wellbeing	Female (*n* = 2709)				Male (*n* = 1873)			
Definitely not (Good Wellbeing)	0.379	0.000	0.322	0.436	0.203	0.000	0.171	0.235
Definitely not (Bad Wellbeing)	0.448	0.000	0.340	0.556	0.282	0.000	0.198	0.366
Probably not (Good Wellbeing)	0.361	0.000	0.336	0.385	0.398	0.000	0.374	0.423
Probably not (Bad Wellbeing)	0.343	0.000	0.302	0.384	0.417	0.000	0.395	0.439
Probably yes (Good Wellbeing)	0.188	0.000	0.155	0.221	0.294	0.000	0.261	0.327
Probably yes (Bad Wellbeing)	0.154	0.000	0.103	0.205	0.231	0.000	0.169	0.292
Definitely yes (Good Wellbeing)	0.072	0.000	0.054	0.091	0.104	0.000	0.083	0.126
Definitely yes (Bad Wellbeing)	0.055	0.000	0.031	0.079	0.070	0.000	0.042	0.099

Source: Author's elaboration on Generations and Gender Survey (GGS) data (Gauthier et al., 2018).

In general, I found that the average fertility intentions score is higher for migrants compared to non-migrants. I also found that both migrants and non-migrants with worse SRH and wellbeing were more likely to give a negative answer to the question “Do you intend to have a/another child during the next three years?” In other words, both migrants and non-migrants with good SRH and wellbeing were more likely to give a positive answer to the question about their short-term fertility intentions. Although a few exceptions to this pattern were found (e.g. male migrant).

Table 1 presents the results of the Blinder-Oaxaca decomposition analysis for the ordinal dependent variable. Statistically significant differences in fertility intentions between migrants and non-migrants were observed across models. The total observed difference in fertility intentions between migrants and non-migrants is 0.6272 for Model 1, 0.5323 for Model 2, 0.5801 for Model 3 and 0.5289 for Model 4 representing 100 % of the observed difference for each model. In other words, on a scale from 1 to 4, the average fertility intentions score is 0.5323 units higher for migrants compared to non-migrants in Model 2, and 0.5289 units higher in Model 4.

The Model 2 and Model 4 are adjusted. Therefore, I am only interpreting these two models here. The different valuation of characteristics (Coef) for migrants in Model 2 contributes 0.2730 units, accounting for 51.3 % of the total difference in omega1. Similarly, the different valuation of characteristics for migrants in Model 4 contributes 53.7 % of the total difference in omega1. Both results are statistically significant.

If migrants had the same characteristics (Char) as non-migrants, their fertility intentions score would be 201.3 % higher in Model 2 and 196.2 % higher in Model 4, contributing to the total difference in omega0, both of which are statistically significant.

Table 2 shows a generalized ordered logistic model for revealing the impact of migration status on fertility intentions of migrants and non-migrants. Among migrants, the probability of definitely not intending to have a child in the next three years is 31.0 %, whereas this probability is 54.4 % for non-migrants. Conversely, migrants have a 9.4 % probability of definitely intending to have a child, whereas non-migrants have only a 3.8 % probability. This pattern has an exception for probably not category of the fertility intention. However, it is not the case for probably yes category of the fertility intention. Among migrants, the probability of probably intending to have a child is 23.1 %, whereas this probability is 11.6 % for non-migrants. All these results are statistically significant.

Table 3 shows a generalized ordered logistic model for fertility intentions of female and male migrants. Among female migrants with good SRH, the probability of definitely not intending to have a child in the next three years is 28.4 %, whereas this probability is 40.4 % for those with poor SRH. The results for wellbeing are similar to those for SRH. Among female migrants with good wellbeing, the probability of definitely not intending to have a child in the next three years is 29.5 %, compared to 42.8 % for those with poor wellbeing.

Female migrants with good SRH have a 16.5 % probability of definitely intending to have a child, whereas those with poor SRH have only a 10.4 % probability. For wellbeing, female migrants with good wellbeing have a 15.8 % probability of definitely intending to have a child, compared to only 9.5 % for those with poor wellbeing. A similar pattern is observed in the "probably yes" and "probably no" categories, with better health associated with more positive intentions to have a child among female migrants.

Among male migrants with good wellbeing, the probability of definitely not intending to have a child is 9.6 %, whereas for those with poor wellbeing, this probability is 12.8 %. Conversely, male migrants with good wellbeing have a 48.5 % probability of definitely intending to have a child, compared to a 40.5 % probability for those with poor wellbeing.

Table 4 shows a generalized ordered logistic model for fertility intentions of female and male non-migrants. Among female non-migrants with good SRH, the probability of definitely not intending to have a child in the next three years is 37.4 %, whereas this probability is 43.1 % for those with poor SRH. The results for wellbeing are similar to those for SRH. Female non-migrants with good wellbeing have a 37.9 % probability of definitely not intending to have a child in the next three years, compared to 44.8 % for those with poor wellbeing.

Female non-migrants with good SRH have a 7.3 % probability of definitely intending to have a child, whereas those with poor SRH have only a 5.9 % probability. Similarly, female non-migrants with good wellbeing have a 7.2 % probability of definitely intending to have a child, compared to only 5.5 % for those with poor wellbeing.

Among male non-migrants with good SRH, the probability of definitely not intending to have a child is 20.4 %, whereas for those with poor SRH, this probability is 22.5 %. Similarly, male non-migrants with good wellbeing have a 20.3 % probability of definitely not intending to have a child, compared to 28.2 % for those with poor wellbeing.

For definitely intending to have a child, male non-migrants with good SRH have a 10.4 % probability, whereas those with poor SRH have a 9.3 % probability. Similarly, male non-migrants with good wellbeing have a 10.4 % probability of definitely intending to have a child, compared to 7.0 % for those with poor wellbeing. A similar pattern, where better health leads to more positive intentions, is observed in the "probably yes" and "probably no" categories for male non-migrants.

## 6 Discussion and conclusion

Researchers in the field of demography have investigated various factors that influence fertility, but the role of perceived health has been little discussed. To address this gap, this study used Poland as an example and examined the connection between different perceived health and intentions regarding fertility. It is probably the first study of its kind to investigate the relationship between perceived health and fertility intentions among migrants and non-migrants of the same nationality at origin together based on two different data sets.

Regarding the first hypothesis (H1), I found it to be true that the relationship between perceived health and intentions to have children among migrant Poles was positively stronger than among non-migrants. This relationship persist in all four models where Model 1 is the crude results of the impact of SRH on fertility intentions, Model 2 is the adjusted results of the impact of SRH on fertility intentions by controlling for age, sex, activity, education and parent status of the respondents. Similarly, Model 3 is the crude results of the impact of wellbeing on fertility intentions, Model 4 is the adjusted results of the impact of wellbeing on fertility intentions by controlling for age, sex, activity, education and parent status of the respondents (see Table 1). The relationship further strengthened by showing the discrete effects of how migration status affects the fertility intentions of both migrants and non-migrants after controlling for SRH, age, sex, activity, education and parent status of the respondents presented in Table 2. It clearly shows a direction that the probability of intending to have a child in the next three years is higher for migrants than that of non-migrants. This finding can be corroborated in existing literature (Alderotti and Trappolini, 2022). Previous studies, including the one conducted by Wallace et al. (2019), have indicated that immigrants in wealthy countries generally enjoy better health compared to native populations in countries of origin. This could be due to several factors, as suggested by various researchers: selective migration (Lee, 1966; McDonald and Kennedy, 2004), cultural influences (Lee et al., 2013), and healthier behaviours among immigrants (Razum et al., 2000). Furthermore, it has been found that poor health is associated with less favourable fertility intentions among migrants, as highlighted in the recent study by Alderotti & Trappolini (2022). When it comes to Polish non-migrants, this study also found a positive relationship between perceived health and intentions to have children.

The main takeaway from the decomposition analysis is the importance of understanding the systemic factors that contribute to the better valuation of characteristics among migrants. The observed differences in characteristics between migrants and non-migrants are expected to some extent, primarily because the average age of migrants is generally lower than that of non-migrants. This age gap may lead to several other differences between migrants and non-migrants.

This study demonstrates a stronger positive association between perceived health and fertility intentions among both men and women, irrespective of their migration status, which partially supports my second hypothesis (H2). As regards migrant women, findings are consistent with an earlier study (Alderotti and Trappolini, 2022) conducted in Italy, which focused exclusively on migrant group and examined the connection between perceived health and fertility intentions. However, I have found no literature exploring the association between perceived health and fertility intentions among non-migrant women and men. Therefore, the positive association between perceived health and fertility intentions among non-migrant women and men cannot be compared to the existing literature.

The positive association between perceived health and fertility intentions among women could be due to the fact that pregnancy places a greater physical and mental burden on women: previous medical research has emphasized the significance of health for women's fertility (Dow and Kuhn, 2004; Katz, 2006). Similarly, men who see themselves as healthy are more likely to feel capable of providing physical, emotional, and financial support to their partner during pregnancy and beyond. This sense of capability can enhance their readiness and desire to have children. On the other hand, men who perceive their health as poor might worry about their ability to support their partner adequately, which can influence their decisions about having children. Moreover, feeling healthy boosts men's confidence in their ability to engage in active parenting. They envision themselves playing with and physically caring for their children, creating strong bonds and participating fully in their child's life. Conversely, men who perceive their health as poor may fear they won't be able to be as involved, which can dampen their desire to become a parent.

This study makes a significant contribution to the existing literature. It is probably the first study to investigate migrants and non-migrants together. I have found that among both migrants and non-migrants, poor perceived health has a considerable negative impact on fertility intentions. I used two health specifications to verify this finding. Therefore, it is highly advisable to consider perceived health as a crucial factor when examining fertility dynamics in these two groups, because different health dimensions can have independent and distinct effects on them. Even after making different profiling (however, I showed results only on one profile) of respondents using several variables that may influence the relationship between perceived health and fertility intentions, such as age, activity, educational level, parent status, reason for migration and duration of staying (last two are only for models on migrants), the association between perceived health and fertility intentions persists.

This study highlights the significance of including perceived health as a determinant of fertility. Given that many European countries are struggling to raise their fertility rates, this investigation is highly relevant. To help migrants and non-migrants achieve their fertility intentions, it may be necessary to provide them with adequate health protection (e.g. by reducing barriers to accessing healthcare services) and improving their living and working conditions. Such an approach could contribute to increasing national fertility levels. This may be especially relevant in countries like Poland, where the fundamental principle of the healthcare system is equity (Dubas-Jakóbczyk et al., 2018), but disparities in healthcare service utilisation still exist (Sowa-Kofta, 2018). The disparity is not limited to non-migrants. Obtaining access to healthcare is among the most significant obstacles that migrants encounter in their destination countries (Buja et al., 2014).

Further investigation is necessary to gain a more comprehensive understanding of the intricacies of the relationship between perceived health and short-term fertility intentions in other settings. One particularly promising avenue of research would be to examine using panel data how short-term fertility intentions translate into actual fertility behaviour.

## 7 Strengths and limitations

This study has some limitations. Firstly, when it comes to the dependent variable, in FPN the possible answers were “definitely not”, “probably not”, “unsure”, “probably yes” and “definitely yes” (5 categories). On the other hand, in GGS the possible answers were “definitely not”, “probably not”, “probably yes” and “definitely yes” (4 categories). Individuals who answered “unsure” were excluded from the analysis to ensure consistency of analysis of the two data sets. The evidence presented in this investigation at least supports the existence of a nexus between perceived health and fertility intentions. Such a connection could potentially mean that poor health has a significant adverse impact on the actual fertility of both migrants and non-migrants. Having said that, this is probably the first study to investigate the relationship between perceived health and fertility intentions among migrants and non-migrants of the same nationality at origin together.

## Declaration of generative AI and AI-assisted technologies in the writing process

During the preparation of this work the author used ChatGPT basic version in order to improve language and readability. After using this basic ChatGPT, the author reviewed and edited the content as needed and take full responsibility for the content of the publication.

## Funding

This work was supported by the 10.13039/501100004442National Science Centre, Poland [grant no 2020/39/D/HS4/02325].

## CRediT authorship contribution statement

**Nasim Ahamed Mondal:** Writing – review & editing, Writing – original draft, Resources, Methodology, Investigation, Formal analysis, Data curation, Conceptualization.

## Declaration of competing interest

The author declare that he has no known competing financial interests or personal relationships that could have appeared to influence the work reported in this paper.

## Figures and Tables

**Table A1:** Frequency distribution of background characteristics of migrants and non-migrants by sex, 2014-15.

Self-rated health and Wellbeing	Migrants				Non-migrants			
	Female		Male		Female		Male	
Characteristics	N*	%⁎⁎	N*	%⁎⁎	N*	%⁎⁎	N*	%⁎⁎
Total	536	100	308	100	2709	100	1873	100
Self-rated health (SRH)								
Good	418	75.4	272	87.9	2,123	78.1	1,538	81.9
Bad	118	24.6	36	12.1	586	22.0	335	18.1
Wellbeing								
Good	452	84	277	90.8	2,589	95.8	1,795	95.8
Bad	84	16	31	9.2	120	4.2	78	4.2
Intention to have a child								
Definitely not	173	38.0	77	26.7	1,616	60.0	869	46.6
Probably not	98	17.9	40	13.2	607	22.1	583	31.0
Probably yes	140	23.4	91	29.3	338	12.2	314	16.7
Definitely yes	125	20.7	100	30.8	148	5.7	107	5.8
Age during interview								
30	241	42.9	114	42.4	657	24.2	509	27.4
31-40	233	38.0	155	42.2	1,097	40.6	712	37.9
>40	62	19.1	39	15.5	955	35.3	652	34.7
Main economic activity								
Employed	326	59.8	238	76.5	1,502.00	55.6	1,278	68.7
Self-employed	122	22.8	50	16.8	611.00	22.5	316	16.8
Unemployed	49	9.9	8	2.7	307	11.3	152	7.7
Others	39	7.5	12	4.1	289	10.7	127	6.9
Education level								
Low (ISCED 0‒2)	99	21.4	110	36.1	164	6.1	157	7.9
Intermediate (ISCED 3‒4)	263	49.0	138	45.0	1,582	58.3	1,224	65.3
High (ISCED 5‒6)	174	29.6	60	19.0	963	35.6	492	26.7
Parent status								
No child	268	49.7	157	50.9	610	21.7	695	37.3
Having a child	268	50.3	151	49.1	2,099	78.3	1,178	62.7
Reasons of migration								
family	165	31.1	15	5.2				
Non-family	371	68.9	293	94.8				
Duration of staying								
Up to 5 years	238	45.6	110	35.8				
5>years	298	54.4	198	64.3				

Sources: Author's elaboration on Families of Poles in the Netherlands (FPN) (Karpinska, et al., 2016). and Generations and Gender Survey (GGS) data (Gauthier et al., 2018). Note:

⁎ N's are unweighted,

⁎⁎ Weighted percentages.

## Appendix

Table A1

## References

[bib0001] Agyemang C., Denktaş S., Bruijnzeels M., Foets M. (2006). Validity of the single-item question on self-rated health status in first generation Turkish and Moroccans versus native Dutch in the Netherlands. Public Health.

[bib0002] Alderotti G., Trappolini E. (2022). Health status and fertility intentions among migrants. International Migration.

[bib0003] Alderotti G., Vignoli D., Baccini M., Matysiak A. (2021). Employment Instability and Fertility in Europe: A Meta-Analysis. Demography.

[bib0004] Ali A.J. (2010). Measuring societal well-being. Competitiveness Review: An International Business Journal.

[bib0005] Allin P. (2007). Measuring societal wellbeing. Econ Lab Market Rev.

[bib0006] Assari S., Lankarani M.M., Burgard S. (2016). Black–white difference in long-term predictive power of self-rated health on all-cause mortality in United States. Ann. Epidemiol..

[bib0007] Barclay K., Kolk M. (2020). The Influence of Health in Early Adulthood on Male Fertility. Popul. Dev. Rev..

[bib0008] Bartel A., Taubman P. (1986). Some Economic and Demographic Consequences of Mental Illness. J. Labor. Econ..

[bib0009] Berrington A., Stone J., Beaujouan E. (2015). Educational differences in timing and quantum of childbearing in Britain: A study of cohorts born 1940–1969. Demogr. Res..

[bib0010] Bhongade M.B., Prasad S., Jiloha R.C., Ray P.C., Mohapatra S., Koner B.C. (2015). Effect of psychological stress on fertility hormones and seminal quality in male partners of infertile couples. Andrologia.

[bib0011] Bloom T.L., Mosher W., Alhusen J., Lantos H., Hughes R.B. (2017). Fertility desires and intentions among U.S. Women by disability status: findings from the 2011–2013 National Survey of Family Growth. Matern. Child Health J..

[bib0012] Boerma T., Hosseinpoor A.R., Verdes E., Chatterji S. (2016). A global assessment of the gender gap in self-reported health with survey data from 59 countries. BMC. Public Health.

[bib0013] Buja A., Fusco M., Furlan P., Bertoncello C., Baldovin T., Casale P., Marcolongo A., Baldo V. (2014). Characteristics, processes, management and outcome of accesses to accident and emergency departments by citizenship. Int. J. Public Health.

[bib0014] Carlsson E. (2022). The realization of short-term fertility intentions among immigrants and children of immigrants in Norway and Sweden. Int. Migr. Rev..

[bib0015] Chandola T., Jenkinson C. (2000). Validating self-rated health in different ethnic groups. Ethn. Health.

[bib0016] Chen J.L., Phillips K.A., Kanouse D.E., Collins R.L., Miu A. (2001). Fertility Desires and Intentions of HIV-Positive Men and Women. Fam. Plann. Perspect..

[bib0017] Cheng J.Y.W., Ng E.M.L. (2007). Body mass index, physical activity and erectile dysfunction: An U-shaped relationship from population-based study. Int. J. Obes..

[bib0018] Cvancarova M., Samuelsen S.O., Magelssen H., Fosså S.D. (2009). Reproduction rates after cancer treatment: experience from the Norwegian radium hospital. J. Clin. Oncol..

[bib0019] d'Albis H., Greulich A., Ponthière G. (2017). Education, labour, and the demographic consequences of birth postponement in Europe. Demogr. Res..

[bib0020] Dantis C., Rizzi E.L. (2020). Transition to first birth during the Great Recession: the case of Greece. Genus..

[bib0021] Dommermuth L., Klobas J., Lappegård T. (2011). Now or later? The Theory of Planned Behavior and timing of fertility intentions. Adv. Life Course Res..

[bib0022] Dow K.H., Kuhn D. (2004). Fertility options in young breast cancer survivors: a review of the literature. Oncol. Nurs. Forum..

[bib0023] Drew S. (2003). Self-Reconstruction and Biographical Revisioning: Survival Following Cancer in Childhood or Adolescence. Health (London).

[bib0024] Dubas-Jakóbczyk, K., Kocot, E., Czerw, A., Juszczyk, G., Karwowska, P., Menne, B., 2018. Health as an investment in Poland in the context of the Roadmap to implement the 2030 Agenda for Sustainable Development and Health 2020.

[bib0025] Fair A., Griffiths K., Osman L.M. (2000). Attitudes to fertility issues among adults with cystic fibrosis in Scotland. Thorax..

[bib0026] Fihel A., Okólski M. (2019). Population decline in the post-communist countries of the European Union. Population Soc..

[bib0027] Fiori F., Rinesi F., Graham E. (2017). Choosing to Remain Childless? A Comparative Study of Fertility Intentions Among Women and Men in Italy and Britain. Eur J Population.

[bib0028] Frisco M.L., Weden M. (2013). Early Adult Obesity and U.S. Women's Lifetime Childbearing Experiences. Journal of Marriage and Family.

[bib0029] Gatta A., Mattioli F., Mencarini L., Vignoli D. (2022). Employment uncertainty and fertility intentions: Stability or resilience?. Popul. Stud. (Camb).

[bib0030] Gauthier A.H., Cabaço S.L.F., Emery T. (2018). Generations and Gender Survey study profile. Longitudinal and Life Course Studies.

[bib0031] Gul-Rechlewicz V. (2020). Immigrants in the Netherlands: Second-class Citizens? The Polish Case. Studia Europejskie - Studies in European Affairs.

[bib0032] Hammoud A.O., Wilde N., Gibson M., Parks A., Carrell D.T., Meikle A.W. (2008). Male obesity and alteration in sperm parameters. Fertil. Steril..

[bib0033] HMHL (2009).

[bib0034] Holton S., Rowe H., Fisher J. (2011). Women's health and their childbearing expectations and outcomes: a population-based survey from Victoria, Australia. Women's Health Issues.

[bib0035] Impicciatore R., Gabrielli G., Paterno A. (2020). Migrants’ Fertility in Italy: A Comparison Between Origin and Destination. Eur J Population.

[bib0036] Jalovaara M., Neyer G., Andersson G., Dahlberg J., Dommermuth L., Fallesen P., Lappegård T. (2019). Education, Gender, and Cohort Fertility in the Nordic Countries. Eur J Population.

[bib0037] Jancewicz B., Markowski S. (2021). Economic turbulence and labour migrants’ mobility intentions: Polish migrants in the United Kingdom, Ireland, the Netherlands and Germany 2009–2016. J. Ethn. Migr. Stud..

[bib0038] Jylhä M. (2009). What is self-rated health and why does it predict mortality? Towards a unified conceptual model. Soc. Sci. Med. (1967).

[bib0039] Kaczmarczyk P., Okólski M. (2008). Demographic and labour-market impacts of migration on Poland. Oxf. Rev. Econ. Policy..

[bib0040] Karpinska K., Dykstra P.A. (2019). Intergenerational ties across borders: a typology of the relationships between Polish migrants in the Netherlands and their ageing parents. J. Ethn. Migr. Stud..

[bib0041] Karpinska K., Dykstra P.A., Fokkema T. (2016). Families of Poles in the Netherlands (FPN) survey. Wave 1. DANS.

[bib0042] Katz P.P. (2006). Childbearing decisions and family size among women with rheumatoid arthritis. Arthritis Care Res. (Hoboken).

[bib0043] Kloc-Nowak W. (2018).

[bib0044] Kohler H.-P., Billari F.C., Ortega J.A. (2002). The Emergence of Lowest-Low Fertility in Europe during the 1990s. Popul. Dev. Rev..

[bib0045] Langeveld N., Stam H., Grootenhuis M., Last B. (2002). Quality of life in young adult survivors of childhood cancer. Support. Care Cancer.

[bib0046] Lappegård T., Rønsen M. (2005). The Multifaceted Impact of Education on Entry into Motherhood. Eur J Population.

[bib0047] Lee E.S. (1966). A theory of migration. Demography.

[bib0048] Lee S., O'Neill A.H., Ihara E.S., Chae D.H. (2013). Change in self-reported health status among immigrants in the United States: Associations with measures of acculturation. PLoS. One.

[bib0049] Lubbers M., Gijsberts M. (2019). Changes in self-rated health right after immigration: a panel study of economic, social, cultural, and emotional explanations of self-rated health among immigrants in the netherlands. Front. Sociol..

[bib0050] Luk B.H.-K., Loke A.Y. (2015). The Impact of Infertility on the Psychological Well-Being, Marital Relationships, Sexual Relationships, and Quality of Life of Couples: A Systematic Review. J. Sex. Marital. Ther..

[bib0051] Matysiak A., Sobotka T., Vignoli D. (2021). The Great Recession and Fertility in Europe: A Sub-national Analysis. Eur J Population.

[bib0052] McDonald J.T., Kennedy S. (2004). Insights into the ‘healthy immigrant effect’: health status and health service use of immigrants to Canada. Soc. Sci. Med. (1967).

[bib0053] McGrath J.J., Hearle J., Jenner L., Plant K., Drummond A., Barkla J.M. (1999). The fertility and fecundity of patients with psychoses. Acta Psychiatr. Scand..

[bib0054] Monga M., Alexandrescu B., Katz S.E., Stein M., Ganiats T. (2004). Impact of infertility on quality of life, marital adjustment, and sexual function. Urology..

[bib0055] Mussino E., Cantalini S. (2022). Influences of origin and destination on migrant fertility in Europe. Popul. Space Place.

[bib0056] Mussino E., Gabrielli G., Ortensi L.E., Strozza S. (2023). Fertility Intentions Within a 3-Year Time Frame: a Comparison Between Migrant and Native Italian Women. Int. Migration & Integration.

[bib0057] Mynarska M., Brzozowska Z. (2022). Things to Gain, Things to Lose: Perceived Costs and Benefits of Children and Intention to Remain Childless in Poland. Soc. Incl..

[bib0058] Mynarska M., Matysiak A., Rybińska A., Tocchioni V., Vignoli D. (2015). Diverse paths into childlessness over the life course. Adv. Life Course Res..

[bib0059] Mynarska M., Wróblewska W. (2017). The health of women of reproductive age and their childbearing intentions. Zdrowie Publiczne i Zarządzanie.

[bib0060] Nijs P., Koninckx P., Verstraeten D., Mullens A., Nicasy H. (1985). Psychological factors of female infertility. Eur. J. Obstet. Gynecol. Reprod. Biol..

[bib0061] Peterson B., Harrell F.E. (1990). Partial proportional odds models for ordinal response variables. J. Roy. Stat. Soc. Ser. C (Appl. Stat.).

[bib0062] Pszczółkowska D. (2024).

[bib0063] Ramlau-Hansen C.H., Thulstrup A.M., Nohr E.A., Bonde J.P., Sørensen T.I.A., Olsen J. (2007). Subfecundity in overweight and obese couples. Human Reprod..

[bib0064] Razum O., Zeeb H., Rohrmann S. (2000). The ‘healthy migrant effect’–not merely a fallacy of inaccurate denominator figures. Int. J. Epidemiol..

[bib0065] Rindfuss R.R., Rindfuss 1946 R.R., Morgan S.P., Swicegood G. (1988).

[bib0066] Schmidt L., Carrell D.T., Peterson C.M. (2010). Reproductive Endocrinology and Infertility: Integrating Modern Clinical and Laboratory Practice.

[bib0067] Schoen R., Astone N.M., Kim Y.J., Nathanson C.A., Fields J.M. (1999). Do fertility intentions affect fertility behavior?. J. Marriage Family.

[bib0068] Sinning M., Hahn M., Bauer T.K. (2008). The Blinder–Oaxaca Decomposition for Nonlinear Regression Models. Stata J..

[bib0069] Sowa-Kofta, A., 2018. ESPN Thematic report on inequalities in access to healthcare Poland.

[bib0070] Statistics Netherlands, S., 2022. How many people immigrate to the Netherlands?

[bib0071] Stonawski M., Potančoková M., Skirbekk V. (2016). Fertility Patterns of Native and Migrant Muslims in Europe. Popul. Space Place.

[bib0072] Tocchioni V. (2018). Exploring the childless universe: Profiles of women and men without children in Italy. Demogr. Res..

[bib0073] Tønnessen M. (2020). Declined Total Fertility Rate Among Immigrants and the Role of Newly Arrived Women in Norway. Eur J Population.

[bib0074] Wallace M., Khlat M., Guillot M. (2019). Mortality advantage among migrants according to duration of stay in France, 2004–2014. BMC. Public Health.

[bib0075] Wanner P., Pecoraro M. (2023). Self-reported health among migrants. Does contextual discrimination matter?. J. Migr. Health.

[bib0076] Wiking E., Johansson S., Sundquist J. (2004). Ethnicity, acculturation, and self reported health. A population based study among immigrants from Poland, Turkey, and Iran in Sweden. J. Epidemiol. Community Health.

[bib0077] Williams R. (2016). Understanding and interpreting generalized ordered logit models. J. Math. Sociol..

[bib0078] Williams R. (2006). Generalized ordered logit/partial proportional odds models for ordinal dependent variables. Stata J..

[bib0079] Woo H., Zajacova A. (2017). Predictive strength of self-rated health for mortality risk among older adults in the United States: Does it differ by race and ethnicity?. Res. Aging.

